# Brucellosis causing bone marrow aplasia in an 11-year-old patient with complete recovery after treatment

**DOI:** 10.1016/j.idcr.2022.e01531

**Published:** 2022-06-15

**Authors:** Nour Youssef, Yolla Youssef, Dolly Noun, Miguel Abboud, Ghassan Dbaibo

**Affiliations:** a Department of Pediatrics and Adolescent Medicine, American University of Beirut Medical Center, Beirut, Lebanon; b Division of Pediatric Infectious Diseases, Department of Pediatrics and Adolescent Medicine, Lebanon; c Center for Infectious Diseases Research, American University of Beirut Medical Center, Beirut, Lebanon

**Keywords:** Brucellosis, Zoonotic infections, Aplastic anemia, Pancytopenia

## Abstract

Brucellosis is one of the most prevalent zoonotic infections in the Middle East. The disease may present with a range of symptoms from a simple febrile illness to severe invasive infections affecting different organ systems (meningitis, osteomyelitis). In this paper we present an eleven-year-old girl who was diagnosed with “idiopathic bone marrow aplasia” and planned for hematopoietic stem cell transplant (HSCT), when pre-transplant work-up showed high brucella titers. The patient was started on doxycycline, rifampin and gentamicin initially, with discontinuation of the latter 3 weeks into therapy. She recovered completely after 8 months of treatment.

## Background

Brucellosis, also known as “Mediterranean fever” or “Malta fever”, is a frequently encountered zoonotic infection in the Middle East. It is usually transmitted to humans from infected animals including cattle, sheep, goats, camels, and pigs via ingestion of food products or by contact with tissue or fluids. It is an important public health concern in many developing countries worldwide [1].

The incidence of brucellosis has been estimated to range from one per 100,000 to 20 per 100,000 in the Middle East and North Africa (MENA) region. This may be an underestimation to actual numbers due to lack of surveillance system among many countries in the region, including Lebanon [2]. A major outbreak was reported in Lebanon in 2017, with the peak of brucellosis cases detected in individuals aged 20–39 years with male predominance. *Brucella melitensis* and *B*. *abortus* are widespread across the MENA region [3].

Brucella infection can be subclinical, however, when present, signs and symptoms are highly variable in the majority of patients including, but not limited to fever, chills, myalgia, malaise, arthralgias, weight loss, headaches, anorexia, abdominal pain, and depression [4]. Physical examination findings are non-specific and may include hepatosplenomegaly and/or lymphadenopathy [5].

## Case presentation

The patient is an 11-year-old Lebanese girl, diagnosed with aplastic anemia. She presented to our center for a second opinion and to discuss treatment with allogeneic hematopoietic stem cell transplant (HSCT). History went back to 1 month prior to presentation when the patient was found to have thrombocytopenia on a routine blood test reaching 60,000/cu.mm. Her platelet count continued to decline, along with her hemoglobin level. She received two doses of intravenous immunoglobulin therapy. Bone marrow aspirate was done and revealed the diagnosis of aplastic anemia. The patient started receiving platelet and packed red blood cell transfusions on a regular basis. Further work up was initiated to plan for a fully matched HSCT from her sister. However, the pretransplant workup showed positive direct and indirect brucella titers (1/160 each). Therefore, she was referred to the Pediatric Infectious Diseases clinic for further evaluation. Upon further questioning, the patient reported a history of headaches, myalgias and polyarticular pain of lower extremities and mid-back, associated with night-time subjective fevers, with no other complaints. There was no recalled history of ingestion of unpasteurized dairy products. Physical exam was remarkable for bilateral knee swelling. Initial blood investigations showed anemia (Hb 7.5 g/dl), leucopenia (white cell count of 2100/cu.mm with an absolute neutrophil count (ANC) of 504/cu.mm), thrombocytopenia (platelet count of 9000/cu.mm) and a reticulocyte percentage of 0.3%. Her liver enzymes were mildly elevated (alanine aminotransferase 178 IU/L) with normal bilirubin and alkaline phosphatase. Lactic dehydrogenase was within normal range. The inflammatory markers were elevated including serum ferritin (1040 ng/ml), erythrocyte sedimentation rate (54 mm/h). C-reactive protein was (1.3 mg/l). The serological tests for HIV, hepatitis C virus, hepatitis B surface antigen, anti-hepatitis B core antigen, and anti-hepatitis B surface antigen were negative. The cytomegalovirus IgG and Epstein–Barr virus IgG were positive, but IgM was negative for both. Antinuclear antibody was negative. Repeated bone marrow aspirate showed a severely aplastic pattern with less than 5% cellular marrow and no evidence of malignancy. The patient was maintained on packed red blood cells (PRBC) and platelet transfusions at a frequency of every 2–3 days. Egg preservation was done as part of HSCT preparation. Blood cultures were collected for brucella assessment that showed no growth and repeated cultures were sterile. Similarly bone marrow culture for brucella was negative for any growth, however, RNA 16 S test of bone marrow aspirate came back positive with *Brucella melitensis*. The patient was started on oral doxycycline 100 mg twice daily and rifampin 600 mg once daily.

Whole body MRI showed numerous bone lesions involving the lower cervical, thoracic, and visualized lumbar spine with low signal intensity on T1, low signal intensity on T2 and faint enhancement after contrast administration. Other few scattered lesions were noted in the left femoral head, bilateral proximal femur, iliac bones and sacrum, and right ulna. Accordingly, Brucella involvement of the bone was presumed, and the patient was admitted to receive intravenous Gentamicin 2.5 mg/kg/dose every 8 h for a total of 3 weeks duration while continuing doxycycline and rifampin. During this period of time, the patient had flares of body aches, at points that correlated with lesions found on MRI, with persistent fatigue. Her hematological parameters improved gradually after treatment initiation. Her last CBCD done 5 months after her last transfusion showed a hemoglobin level of 13.7 g/dl, white cell count of 3600 /cu.mm, and platelet count of 107000 /cu.mm. The patient’s diffuse bony pain improved significantly.

**Fig. 1:**
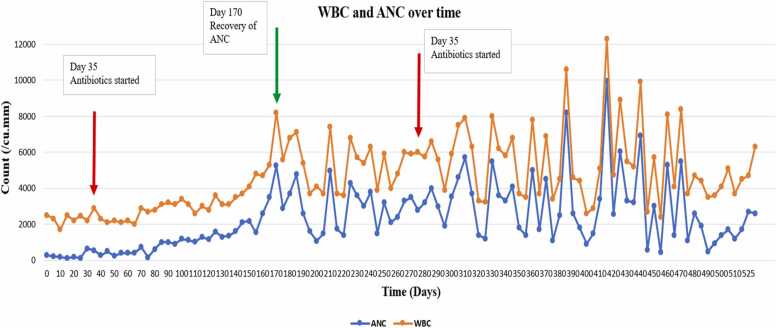
Trends of WBC count and ANC with time (WBC: white blood cell; ANC: absolute neutrophil count).

**Fig. 2:**
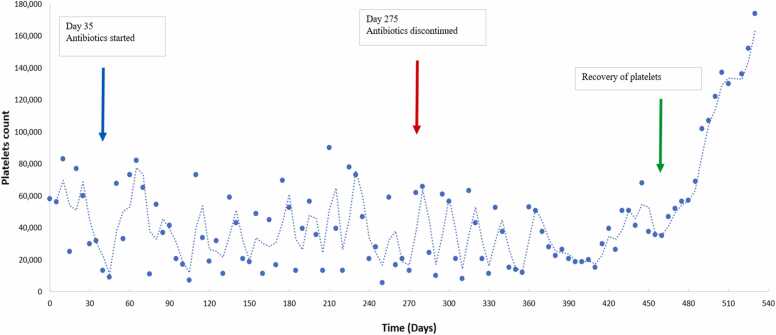
Platelet count before and after treatment. Graph representing the trend of platelet count from the time of diagnosis to the post treatment period.

The treatment regimen consisted of 21 days of IV Gentamicin, along with a total of 8 months of oral doxycycline and rifampin. We observed as expected, a late platelet recovery after almost 14 months from initiation of antibiotics. On the contrary, WBC and ANC values trended up after completing 4 months of antimicrobial therapy. This is demonstrated in Fig. 1, Fig. 2 respectively. It is important to note that the need to transfuse our patient started to decrease gradually at almost 2 months post antibiotic initiation.

To our knowledge, this is the first case of a brucella infection causing aplastic anemia in Lebanon.

## Discussion

Brucellosis is an endemic zoonotic disease in most countries of the MENA region. For example, in Saudi Arabi, the incidence of brucellosis is estimated to be 70 per 100,000 people/year [6]. Up to 30% of the reported cases are children [7]. Although the incidence rate of brucellosis has been trending down in developed countries, it remains to be a major health problem in developing ones. Other countries have variable seroprevalence rates ranging from 5% in Lebanon to 8% and 12% in Jordan and Kuwait, respectively [8].

Brucellosis may present with a range of symptoms that may involve one or multiple organ systems [9]. The most common symptom is fever. Children with brucellosis often have hematologic abnormalities including anemia, thrombocytopenia, and pancytopenia [10].

In the literature, a three-year-old boy was reported to have secondary hemophagocytic lymphohistiocytosis associated with brucellosis [11]. In another paper by Makis et al., a case of a 5-year-old girl with *Brucella melitensis* infection presenting with severe thrombocytopenic purpura was described. Treatment included intravenous immunoglobulin and a combination of antibiotics [12]. In Saudi Arabia, a series of 115 children with brucellosis was reported, four of whom had severe thrombocytopenia. All four patients were successfully treated with antimicrobial agents [13]. In Lebanon, Farah et al. reported a case of severe thrombocytopenic purpura as the presenting symptom of brucellosis in an 8-year-old boy [14].

A prospective study was conducted in Iran to study the relative frequency of pancytopenia in patients with brucellosis. Results showed that 18.3% of enrolled patients had pancytopenia at diagnosis. Interestingly, all patients grew *Brucella melitensis* in their blood cultures [15].

Bone marrow hypoplasia/apalsia secondary to brucellosis has been rarely described previously [16]. Shayib and Aysha et al. have described association of brucella with severe bone marrow hypocellularity [17]. In Turkey, an 11-year-old boy was reported to have bone marrow aplasia that was attributed to brucellosis. The patient’s pancytopenia recovered completely after 6 weeks of antimicrobial treatment [18].

In our patient, the bone marrow aplasia was so severe it almost led to HSCT, had the positive brucella titers not been detected or requested in the pre-transplant work up. *Brucella melitensis* infection was confirmed only by detection with the 16 S RNA assay on the bone marrow sample whereas blood and bone marrow cultures were negative. It is unknown what the outcome of transplantation of this patient would have been, but it is likely that it would have failed without treatment for brucellosis. Our patient fully recovered after 8 months of treatment.

The most focal form of brucellosis is osteoarticular disease, involving peripheral arthritis, sacroiliitis and spondylitis, occurring in 70% of infected patients [19]. Our patient had numerous bone lesions involving the lower cervical, thoracic, and lumbar spine most likely due to osteoarticular involvement with brucella infection. The patient reported worsening of her joints and thoracic region pain few weeks into treatment likely related to the inflammatory response to killed Brucella organisms.

Complications of brucellosis can involve multiple organ systems and occur more frequently in adults than in children [4].

Treatment of human brucellosis has been well described being optimally a dual or triple antimicrobial regimen for a prolonged period of time [20] The optimal combination includes rifampicin combined with either doxycycline or a fluoroquinolone (in adults) or trimethoprim-sulfamethoxazole (in children younger than 8 years of age) [21]. Several reports in the literature advocate for adding an aminoglycoside being streptomycin or gentamicin for the first 2–3 weeks of treatment, particularly in complicated brucellosis [21]. The total duration and choice of therapy are usually related to the patient’s characteristics, extent and severity of the disease, where the duration is usually longer in aggressive disease with either CNS or bone involvement. Treatment courses below 1 month have been associated with an increased rate of treatment failure. Our patient received a long course of 8 months, owing to the slow improvement, and the persistent need for frequent transfusions early during the treatment course. Her 1-year follow-up post remission is unremarkable with normal blood tests and growth patterns.

## Conclusion

Brucellosis may present with a wide range of symptoms with serious morbidities such as bone marrow suppression in immunocompetent patients living in or visiting countries endemic for the infection. Pre-transplant work up for aplastic anemia or bone marrow failure should include screening for brucellosis in endemic countries. A high index of suspicion, early diagnosis, and appropriate therapy are of paramount importance in proper recovery and improved outcome.

## Funding

This research did not receive any specific grant from funding agencies in the public, commercial, or not-for-profit sectors.

## CRediT authorship contribution statement

N.Y and Y.Y wrote the initial draft of the manuscript. All authors read, edited, and approved the final version of the manuscript.

## Declaration of Competing Interest

The authors declare that they have no known competing financial interests or personal relationships that could have appeared to influence the work reported in this paper.
